# Tumor Microenvironment Modulates Invadopodia Activity of Non-Selected and Acid-Selected Pancreatic Cancer Cells and Its Sensitivity to Gemcitabine and C18-Gemcitabine

**DOI:** 10.3390/cells13090730

**Published:** 2024-04-23

**Authors:** Tiago M. A. Carvalho, Madelaine Magalì Audero, Maria Raffaella Greco, Marilena Ardone, Teresa Maggi, Rosanna Mallamaci, Barbara Rolando, Silvia Arpicco, Federico Alessandro Ruffinatti, Alessandra Fiorio Pla, Natalia Prevarskaya, Tomas Koltai, Stephan J. Reshkin, Rosa Angela Cardone

**Affiliations:** 1 Department of Biosciences, Biotechnology and Environment, University of Bari, 70125 Bari, Italy; tiagomac94@gmail.com (T.M.A.C.); grecoraffaella@hotmail.it (M.R.G.); marilena.ardone@uniba.it (M.A.); teresa.maggi3@gmail.com (T.M.); rosanna.mallamaci@uniba.it (R.M.); stephanjoel.reshkin@uniba.it (S.J.R.); 2 U1003 PHYCEL Laboratoire de Physiologie Cellulaire, Inserm, University of Lille, 59000 Lille, France; madelaine.audero@gmail.com (M.M.A.); alessandra.fiorio@unito.it (A.F.P.); natacha.prevarskaya@univ-lille.fr (N.P.); 3 Department of Drug Science and Technology, University of Turin, 10125 Turin, Italy; barbara.rolando@unito.it (B.R.); silvia.arpicco@unito.it (S.A.); 4 Laboratory of Cellular and Molecular Angiogenesis, Department of Life Sciences and Systems Biology, University of Turin, 10123 Turin, Italy; federicoalessandro.ruffinatti@unito.it; 5 Hospital del Centro Gallego de Buenos Aires, Buenos Aires 2199, Argentina; tkoltai@hotmail.com

**Keywords:** hypoxia, cell invasion, extracellular acidosis, invadopodia, chemoresistance

## Abstract

Background: Pancreatic ductal adenocarcinoma (PDAC) is a deadly disease with high mortality due to early metastatic dissemination and high chemoresistance. All these factors are favored by its extracellular matrix (ECM)-rich microenvironment, which is also highly hypoxic and acidic. Gemcitabine (GEM) is still the first-line therapy in PDAC. However, it is quickly deaminated to its inactive metabolite. Several GEM prodrugs have emerged to improve its cytotoxicity. Here, we analyzed how the acidic/hypoxic tumor microenvironment (TME) affects the response of PDAC cell death and invadopodia-mediated ECM proteolysis to both GEM and its C18 prodrug. Methods: For this, two PDAC cell lines, PANC-1 and Mia PaCa-2 were adapted to pH_e_ 6.6 or not for 1 month, grown as 3D organotypic cultures and exposed to either GEM or C18 in the presence and absence of acidosis and the hypoxia inducer, deferoxamine. Results: We found that C18 has higher cytotoxic and anti-invadopodia activity than GEM in all culture conditions and especially in acid and hypoxic environments. Conclusions: We propose C18 as a more effective approach to conventional GEM in developing new therapeutic strategies overcoming PDAC chemoresistance.

## 1. Introduction

Pancreatic ductal adenocarcinoma (PDAC) is one of the most aggressive and lethal tumors and has a dismal prognosis, where only around 9–11% of the patients survive for 5 or more years [1]. It is expected to become the second leading cause of cancer-related deaths in the next decades [2]. Many patients already have extraregional metastasis at the time of diagnosis and up to 70% of patients die from metastasis formed very early [3]. Since cancer cells’ dissemination to distant sites is the primary cause of cancer mortality, the need for new therapeutic modalities targeting metastasis is critical.

Several biological steps are involved in cancer cell invasion, including cell adhesion, motility and the proteolytic remodeling of the extracellular matrix (ECM) [4]. ECM degradation is one of the most important steps of cancer cell invasion and is driven by the activity of invadopodia [5]. Invadopodia are actin-rich plasma membrane structures utilized by cancer cells to degrade the ECM, in order to invade the surrounding tissues [5]. The activity of these cell protrusions relies on the coordination of an F-actin–cortactin core surrounded by actin regulatory proteins, such as ARP2/3, N-WASP, cofilin, and actin cross-linking proteins. Scaffolding and adhesion proteins together with ion channels are required for invadopodia stabilization: the constant delivery of invadopodial proteases in these structures and their ECM proteolytic ability, which ensures cancer cells’ penetration in the matrix [6,7,8,9]. It is now well-recognized that invadopodia drive invasion and metastasis, making the interpretation of their dynamics important to design efficient anti-metastatic therapies [10].

The PDAC local tumor microenvironment (TME), where tumors occur and develop, consists of a complex arrangement of ECM, fibroblasts, stellate cells, and immune cells [11]. The TME of PDAC is characterized by an abundant desmoplastic reaction, responsible for a dense fibrotic stroma [12], which hinders blood supply-generating areas of hypoxia and extracellular acidosis in the tumor milieu. The hypoxic/acidic TME promotes immunosuppression and the expansion of more aggressive tumor clones, including both cells endowed with exuberant invasive abilities and cells with stemness properties such as cancer stem cells (CSCs) [12]. Characteristics related with TME such as cell plasticity, heterogeneity of the tumor, arrangement of the tumor stroma, epithelial-to-mesenchymal transition (EMT), reprogrammed metabolism, acidic extracellular pH (pH_e_), and hypoxia can strongly impact therapy outcomes [12]. Indeed, several studies have shown that the different TME cell populations and their released cytokines are involved in the control of the proliferation, metastasis, and chemoresistance of pancreatic cancer cells [13,14,15].

Gemcitabine (GEM), a deoxycytidine nucleoside analog, is the gold standard treatment for advanced PDAC since 1997 [16]. The mechanism of action of GEM relies on two metabolites: GEM 3-phosphate, which interferes with tumor growth through its incorporation into DNA, and GEM diphosphate, which disrupts DNA synthesis and tumor growth by inhibiting the ribonucleotide reductase [17]. Nonetheless, GEM therapy only confers a minimal survival advantage to PDAC patients, in part due to its short in vivo half-life [18]. In fact, one of its main disadvantages is the rapid deamination to its inactive metabolite, 2′,2′-difluorodeoxyuridine, by cytidine deaminase. Therefore, several lipophilic prodrugs have been developed and tested through the linkage of the 4-amino group of GEM with valeroyl, heptanoyl, lauroyl, and stearoyl linear acyl derivatives to increase its stability and bioavailability [19]. The activity of GEM prodrugs has been successfully studied in several type of tumors [18,20,21], including PDAC [22], where we previously demonstrated that one of the tested GEM prodrugs, 4-(N)-stearoyl-gemcitabine (C18), can represent a more effective therapy against the highly chemoresistant PDAC cells. Indeed, C18 demonstrated a higher efficacy in reducing growth and increasing cell death and apoptosis compared to GEM in PDAC cells in a platform of three-dimensional (3D) organotypic cell cultures, taking into account the changing stromal/ECM composition during PDAC progression [22]. However, both the drug delivery and the activity of the chemotherapeutic agent are strongly dependent on the TME, which represents a physical barrier that limits in vivo drug targeting and efficacy [12,23,24]. Furthermore, both the physiology and therapeutic sensitivity of cancer cells may be modified by adaptation to the TME and especially to the acidic component of the TME [25]. Indeed, we recently demonstrated that the acidic growth condition selects for more aggressive and invasive PDAC cells in vitro [26]. Therefore, it is crucial to study drug efficacy in the context of the stromal, acidic, and hypoxic TME and apply in vitro methodologies to study their efficacy not only against the growth of the primary tumor but also in counteracting invasion and metastasis.

In this study, we used 3D organotypic cultures of two different pancreatic cancer cell lines, cultured on a mix of Matrigel and Collagen I ECM, in order to mimic the in vivo interactions between tumor cells and their dynamic, stromal TME. Whereas Matrigel mimics an early tumor stage, a Collagen I-rich ECM more likely reflects the desmoplastic reaction of the advanced PDAC. Using this model, we first evaluated the efficacy of GEM and C18 in blocking tumor growth and inducing cell death at either pH_e_ 7.4 or 6.7 in PDAC cells that have been acid selected or not. Furthermore, as cancer patient mortality is mainly due to metastasis, we assessed the contribution of acidosis and hypoxia to the invadopodia activity of these PDAC cell lines and the relative potential of both drugs (GEM and C18) to inhibit this pro-metastatic process also under acidic and hypoxic conditions.

## 2. Materials and Methods

### 2.1. Cell Culture and pH Selection

Two parenchymal PDAC cell lines, the PANC-1 and MiaPaCa-2 cell lines, were used to perform all the described experiments. Both cell lines were maintained in complete RPMI 1640 (Gibco, Life Technologies, Carlsbad, CA, USA, cat #11875-093) supplemented with 10% fetal bovine serum (FBS, Gibco, Life Technologies; cat #10270-106), 50 µg/mL gentamycin (Gibco, Life Technologies, Carlsbad, CA, USA), and 1% penicillin–streptomycin solution (Gibco, Life Technologies; Carlsbad, CA, USA cat #15140-122) and were cultured at 37 °C in humidified air containing 5% CO_2_. pH_e_-selected cells were generated after 1 month exposure to acidic medium (pH_e_ 6.6) before performing the experiments as described in [26]. Experiments requiring pH_e_-selected cells were performed in a time frame of maximum 2 weeks following recovery to pH_e_ 7.4. Both the pH_e_ 7.4 and pH_e_ 6.7 medium were refreshed every two days. For pH adjustments of the cell culture media to pH_e_ 6.6 and pH_e_ 6.7, the complete RPMI 1640 was complemented with NaHCO_3_ according to the Henderson–Hasselbalch equation to derive the desired pH_e_ as previously described [26].

### 2.2. Drug and Prodrug Stability

Gemcitabine (GEM) was purchased from Accord Healthcare (Milan, Italy) and the GEM lipophilic prodrug (C18) was synthesized according to Immordino et al. [19]. The stability of GEM and C18 were evaluated in complete RPMI 1640 cell culture media under three different pH conditions; the pH was corrected to 6.7 and 7.4, used for the cell culture, and also to pH 5.0. The stability assessment was conducted similarly to Jansen et al. [27]. Briefly, 1 mg/mL solution of GEM or C18 in DMSO was added to RPMI 1640 (with pH corrected to 5.0, 6.7, or 7.4), to obtain the 50 μg/mL final concentration in glass tubes. The resultant solutions were incubated at 37 ± 0.5 °C; after 0, 24, 48, 72, and 96 h, 300 µL of reaction mixtures were withdrawn and added to 300 µL of CH_3_CN containing 0.1% TFA. The samples were vortexed and centrifuged for 5 min at 2500× *g*. The clear supernatant was filtered by 0.45 µm PTFE (Alltech, Upland, CA, USA) and analyzed by RP-HPLC as described in [22]. The RP-HPLC procedure allowed the quantification of the GEM and C18 prodrug and the qualitative analysis of any degradation products. Calibration curves of GEM and C18 were obtained with standard solutions of compounds (r^2^ > 0.99); seven calibration standards (1, 5, 10, 20, 25, 50, 100 µg/mL) were prepared by dilution from 1 mg/mL stock solutions of compounds in a mixture of CH_3_CN/water/TFA (50/50/0.1 *v*/*v*/*v*).

### 2.3. Three-Dimensional Organotypic Culture

Organotypic 3D cultures were produced as previously reported [7,8,9]. Briefly, Matrigel (Corning Matrigel Growth Factor Reduced Basement Membrane Matrix, Phenol Red-Free, cat #430663, Rodano, Italy) was diluted in RPMI 1640 without gentamicin sulfate and without FBS to a final concentration of 7 mg/mL. Collagen I bovine (Thermo Fisher Scientific, cat #A1064401, Rodano, Italy) was diluted to the final concentration of 3 mg/mL according to the manufacturer’s directions. A mixture of the resultant solutions of Matrigel and Collagen I was produced with the final proportion of 90% Matrigel and 10% Collagen. In total, 100 µL of this mixture was added to the bottom of a 96 well-plate and then incubated at 37 °C with 5% CO_2_ for 1 h, allowing the mixture to create a thin layer on the bottom of the wells. The cells were then seeded in each well on the top of this extracellular matrix-mimicking layer.

### 2.4. Cell Death Ethidium Homodimer Assay

Cell death was assessed using the cell-impermeant death indicator ethidium homodimer-1 (Thermo Fisher Scientific; cat # E1169, Rodano, Italy). The high-affinity nucleic acid stain emits red fluorescence only when bound to the DNA of dead cells. Cells were seeded in 96-well plates and cultured in the above-described organotypic 3D model. After 24 h, cell lines were treated with 50 μM of GEM or C18 in combination with 16 nM of ethidium homodimer, added directly in the cell medium. Cell growth and cell death were monitored in the following days for 96 h. A Nikon Inverted Microscope Eclipse Ti-S at 4× magnification was utilized to capture images and then analyzed using Image J 1.46r software (Wayne Rasband, NIH, USA, http://imageJ.nih.gov/ij, accessed on 10 December 2023).

### 2.5. Cell Viability Assay

Cell viability was analyzed by using the Resazurin Cell Viability Assay Kit (Abcam; cat # Ab129712, Milano, Italy), following the manufacturer’s protocol. Briefly, cells were seeded in 96-well plates on top of the extracellular matrix gel prepared as described above. After 24 h, cells were incubated with GEM or C18 for 96 h. At the end of the treatment, 15 μL of resazurin was added directly in the medium of each 96-well for 3 h at 37 °C and the fluorescent signals were detected at Ex/Em 535/590 nm by using a Varian Cary Eclipse Fluorescence Spectrophotometer (Agilent Technologies, Santa Clara, CA, USA). For each treatment, the obtained data were normalized with the respective control group.

### 2.6. Fluorescent Matrigel Layer Preparation and Invadopodia Activity Assay

Invadopodia digestive activity was measured as previously described [7,8,9,26]. Briefly, Quenched BODIPY linked to BSA (DQ-Green-BSA; Thermo Fisher Scientific, Invitrogen™ cat #D12050, Rodano, Italy) was added to a final concentration of 30 µg/mL to the 90% Matrigel and 10% Collagen I matrix mix described above in Section 2.3. This was used to cover 12 mm round glass coverslips placed in the bottom of a 24-well plate and allowed to polymerize for 30 min in a humidified incubator at 37 °C. Then, 30,000 cells/coverslip were seeded in the top of the polymerized matrix at both physiological (pH_e_ 7.4) and acidic (pH_e_ 6.7) conditions. One hour after cell seeding, cells were either treated or not with the gemcitabine drugs or deferoxamine (DFX) and incubated overnight. After extensive washing with PBS to remove suffering/dying cells, the slides were fixed with 3.7% paraformaldehyde in PBS and processed for immunofluorescence. Invadopodia-dependent ECM digestion was evaluated microscopically in which focal proteolysis produces green fluorescence on a black background, which is used to quantitatively measure the proteolytic activity level. The quantity of invadopodia activity was determined with the following measurements: (i) percentage of cells with active invadopodia, (ii) number of invadopodia per active cell, and (iii) pixel density of digestion performed by individual invadopodia. The mean total actual invadopodia proteolytic activity per 100 cells was calculated as follows: invadopodial index = percentage of invadopodia-positive cells × mean pixel density of invadopodia/cell.

### 2.7. Statistical Analysis

Statistical significance of differences between experimental groups was evaluated by unpaired *t*-test with Welch’s correction or one-way ANOVA, followed by Tukey’s post-test, using GraphPad Prism v6.01 (GraphPad Software, https://www.graphpad.com/ accessed on 10 December 2023). All experimental data are shown as mean ± SEM and significant differences were considered when *p*-values < 0.05.

## 3. Results

### 3.1. GEM and C18 Stability in Culture Media

We first evaluated the chemical stability of GEM and its C18 prodrug in complete RPMI 1640 cell culture media under three pH conditions: 6.7 and 7.4, to be used for the cell culture experiments and also pH 5.0, to have the highest possible acidity condition (Supplementary Figure S1A,B).

Both compounds showed good stability for 96 h in cell culture media under the three different pH conditions. The percentage of unmodified GEM after 96 h was found to be approximately 99% at pH 7.4 and 6.7. Even at pH 5, an acidic condition known to reduce GEM chemical stability [27], the percentage of unmodified GEM was higher than 97%. The C18 prodrug proved to be slightly less stable, with 95% to 97% of unmodified C18 at all the pHs. Interestingly, C18 was more stable at pH 5.0 than the other pHs, with the percentage of unmodified C18 after 96 h being 97%. Importantly, the only C18 degradation product observed was GEM.

### 3.2. C18 Is More Effective than GEM in Reducing Cell Viability and Increasing Cell Death at Both Physiological and Acidic Extracellular pH

We have previously determined the effect of GEM and C18 on PDAC cell viability and death when cells are cultured in 3D on different ECM compositions and treated with both drugs at a pH_e_ of 7.4 [22]. However, the pH_e_ value of 7.4 does not reflect the interstitial acidosis of an advanced tumor. For this, we compared the cytotoxic effect of C18 compared to GEM in two different PDAC cell lines with different genetic backgrounds, Panc-1 and MiaPaCa-2, that were cultured either at the physiological pH_e_ of 7.4 or cultured for 1 month at pH_e_ 6.6, in order to develop an acid-selected phenotype [26]. Both the non-selected cells and the acid-selected cells were grown as 3D organotypic cultures of 90M-10C and treated for 96 h with 50 µM of the two drugs at both pH_e_ 7.4 and pH_e_ 6.7.

#### 3.2.1. Cell Viability

**Figure 1:**
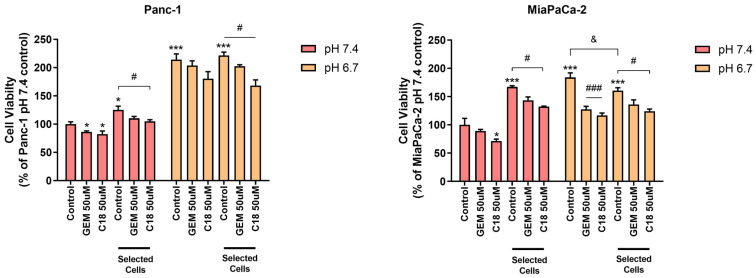
Cell viability of Panc-1 cells and MiaPaCa-2 cells treated with 50 μM of GEM or C18 for 96 h in 90% Matrigel/10% Collagen I 3D ECM. C18 was more effective in reducing cell viability of both cell lines at both pH_e_s compared to GEM. Cell viability was measured by Resazurin Cell Viability Assay, as described in Section 2. Error bars indicate mean ± S.E.M. of at least three independent experiments. Statistics legend: *p* < 0.05 (*) or *p* < 0.001 (***), compared control of non-selected cells at pH_e_ 7.4; *p* < 0.05 (#) or *p* < 0.001 (###); *p* < 0.05 (&) compared to internal control.

As shown in Figure 1, at pH_e_ 7.4 and in control conditions, both acid-selected cell lines increased their cell viability compared to non-selected cells and this increase was somewhat more pronounced for the MiaPaCa-2 cells. Furthermore, cell viability in both the non-selected cell lines was also stimulated by a short-term (4 days) exposure to extracellular acidosis (pH_e_ 6.7), while only Panc1-selected cells increased their cell viability compared to their acid-selected counterparts at pH_e_ 7.4. Importantly, the reduction in cell viability by C18 was always stronger than by GEM at both pH_e_s and in both acid-selected and non-selected cells.

#### 3.2.2. Cell Death

**Figure 2:**
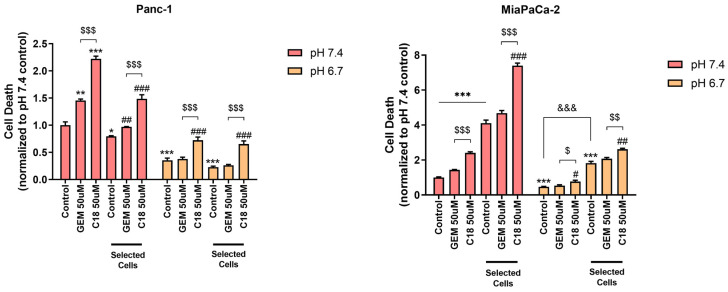
Cell death of Panc-1 cells and MiaPaCa-2 cells treated with 50 μM of GEM or C18 for 96 h in 90% Matrigel/10% Collagen I 3D ECM. C18 caused a higher increase in cell death of both cell lines compared to GEM in all the culture conditions. Cell death was assessed by using the cell-impermeant death indicator ethidium homodimer-1, as described in the Section 2. Error bars indicate mean ± S.E.M. of at least three independent biological replicates. Statistics legend: *p* < 0.05 (*), *p* < 0.01 (**) or *p* < 0.001 (***), when compared to pH_e_ 7.4 control of non-selected cells; *p* < 0.05 (#), *p* < 0.01 (##) or *p* < 0.001 (###), when compared to the respective control; *p* < 0.001 (&&&) as indicated by bar in figure; *p* < 0.05 ($); *p* < 0.01 ($$) or *p* < 0.001 ($$$) as indicated by bar in figure.

As shown in Figure 2, in Panc-1 control cells, we found that the acid-selected cells had a decreased death compared to the non-selected cells at both pH_e_s (~1.25- and 1.60-fold decrease for pH_e_ 7.4 and pH_e_ 6.7, respectively), while in MiaPaCa-2 cells (right graph), acid pH selection increased basal cell death compared to the non-selected cells at both pH_e_s (~4.1- and 3.8-fold increase for the non-selected and pH-selected cells, respectively). Moreover, a short-term exposure to the acidic environment decreased cell death in both cell lines and in both the non-selected cells and the acid-selected cells (2.85- and 3.60-fold in Panc-1 and 2.13- and 2.25-fold in MiaPaCa-2 at pH_e_ 7.4 vs. pH_e_ 6.7, respectively, for non-selected and acid-selected cells), suggesting that extracellular acidosis protects the cells from cell death independently of the acidic-driven selection. Importantly, in both control and acid-selected cells of both cell lines, C18 was much more cytotoxic than GEM in all the experimental conditions (~2- to 3-fold increase in all culture conditions).

### 3.3. Tumor Cell ECM Degradation Is Increased by Hypoxia at Both pH_e_ 7.4 and pH_e_ 6.7 and Decreased in Normoxia at pH_e_ 6.7

The TME acts as a selective pressure mechanism on cancer cells, favoring tumor growth and survival of the most aggressive clones, contributing to drug resistance and metastatic behavior [28]. As the extracellular acidic and hypoxic conditions of the TME promote malignant progression and stimulate the invasion of more aggressive cancer cells [12], we next assessed whether both hypoxia and extracellular acidosis could promote the proteolytic degradation of the ECM in both Panc-1 and MiaPaCa-2 cells. For this, both cell lines were exposed overnight to pH_e_s of 7.4 and 6.7 in combination with either 100 µM or 200 µM deferoxamine (DFX), a chemical inducer of the hypoxic phenotype.

As reported in Supplementary Figure S2, the mean ECM proteolytic activity/cell for both cell lines was dose-dependently stimulated by DFX at both pH_e_s. Interestingly, while the percentage of cells able to degrade the ECM in Panc-1 cells did not change by exposure to acidic pH_e_ in normoxic conditions, it increased in the presence of hypoxia at both pH_e_ 7.4 and 6.7. On other hand, in MiaPaCa-2 cells, the percentage of digestive-positive cells was not significantly altered after their exposure to either extracellular acidosis or hypoxia.

**Figure 3:**
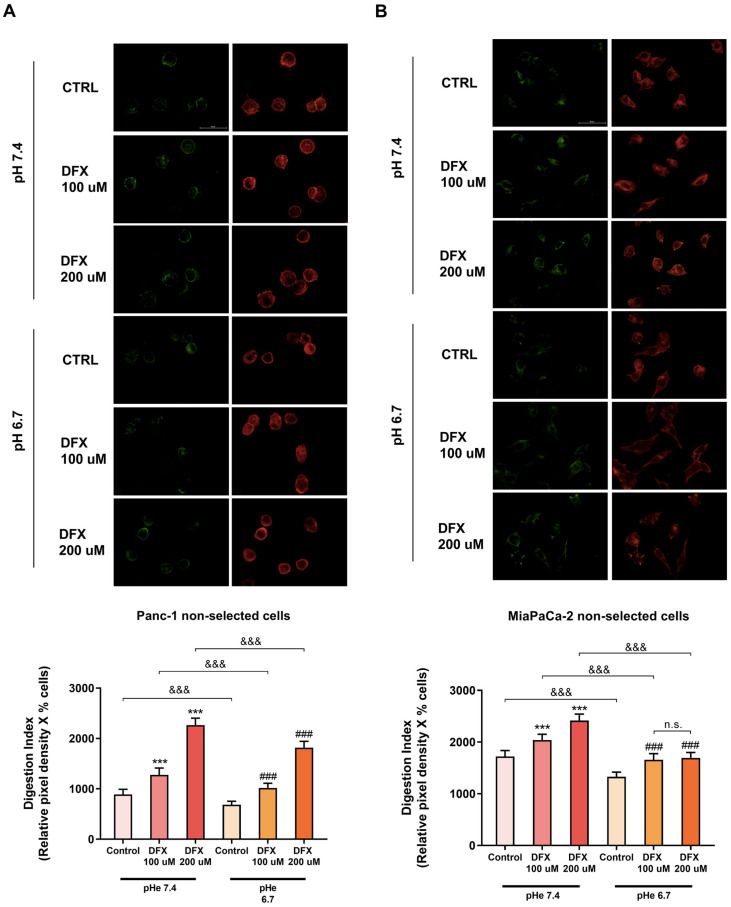
Effect of both hypoxia and acidic pH_e_ on the invadopodia activity of non-acid-selected Panc-1 cells (**A**) and MiaPaCa-2 cells (**B**). The upper panels show typical images of proteolytic digestion in green and actin in red. The white bar in the upper right (CTRL) panel of each cell line represents 50 µm. The resulting histograms (lower panels) show that hypoxia increased the Digestion Index of both cell lines, while extracellular acidosis caused the opposite effect. The percentage of cells that formed invadopodia and their ECM degradation was determined by fluorescence microscopy. The mean total invadopodia proteolytic activity was then calculated as follows: Digestion Index = percentage of invadopodia-positive cells × mean pixel density of invadopodia/cell. Error bars indicate mean ± S.E.M. (*n* = 3). *** *p* < 0.001 when compared with control pH_e_ 7.4; ### *p* < 0.001 when compared with control pH_e_ 6.7; &&& *p* < 0.001 when compared within the same treatment between the two different pH_e_. n.s. = not significant.

When the cellular Digestion Index was calculated as the product of the percentage of ECM-digesting positive cells for the mean ECM digestion/cell (Figure 3A,B), we found that hypoxia and acidic pH_e_ had opposite effects on the invadopodia-mediated ECM proteolysis of both cell lines. Indeed, while the Digestion Index was decreased by exposure to pH_e_ 6.7 in normoxic conditions, as previously reported [26], it was dose-dependently enhanced by hypoxia at both pH_e_s.

### 3.4. Both Hypoxia and pH_e_ Increases Alone and Synergistically the ECM Degradation in the pH-Selected Cells

As the PDAC pH-selected cells exhibit a higher cell viability than the non-selected cells [26], we next evaluated whether hypoxia and extracellular acidosis also influenced the ECM-digesting ability of these cells in comparison to the non-selected cells.

**Figure 4:**
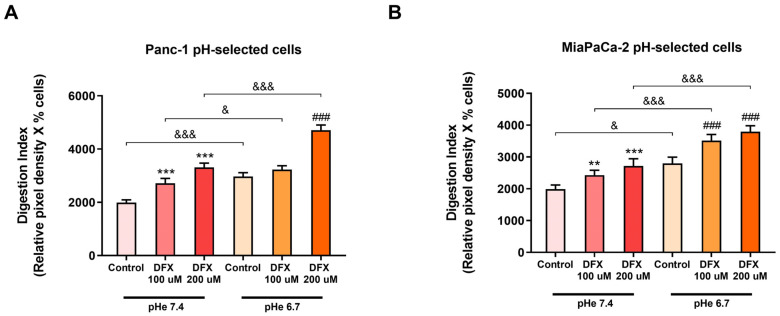
Effect of both hypoxia and acidic pH_e_ on the invadopodia activity of Panc-1 pH-selected cells (**A**) and MiaPaCa-2 pH-selected cells (**B**). Hypoxia and acidosis increased the Digestion Index of both cell lines and together enhanced the proteolytic activity in a synergistic manner. The percentage of invadopodia-positive cells and their ECM degradation was determined by fluorescence microscopy. The mean total invadopodia proteolytic activity was then calculated as follows: Digestion Index = percentage of invadopodia-positive cells × mean pixel density of invadopodia/cell. Error bars indicate mean ± S.E.M. (*n* = 3). ** *p* < 0.01; *** *p* < 0.001 when compared with control pH_e_ 7.4; ### *p* < 0.001 when compared with control pH_e_ 6.7; & *p* < 0.05 and &&& *p* < 0.001 when compared within the same treatment between the two different pH_e_.

As can be seen in Figure 4, the invadopodial Digestion Index of the acidic pH-selected cells of both cell lines was stimulated by both extracellular acidosis and the increasing concentration of DFX at both pH_e_s. Importantly, the maximum Digestion Index was obtained when the two cell lines were simultaneously exposed to both the TME conditions, leading to an additive increase in the ECM proteolytic activity. These increases in the Digestion Index by DFX-induced hypoxia and extracellular acidosis were due to the increase in the proteolytic digestion of the ECM/cell rather than the percentage of ECM-digestive cells (Supplementary Figure S3).

### 3.5. C18 Is More Effective than GEM in Counteracting the DFX-Induced Hypoxic Stimulation of Proteolytic Activity and Drug Resistance in the Non-Acid-Selected Cells

Despite the promising results of C18 in inducing PDAC cell death, it is important to determine whether this prodrug is also more effective than GEM in reducing the mechanisms involved in metastatic process, such as the invadopodia-mediated ECM digestion. To explore this, we first cultured both PDAC non-acid-selected cell lines in the absence and presence of hypoxia and exposed them overnight to 50 µM of both GEM and C18. Typical images of the proteolysis and actin cytoskeleton are shown in Supplementary Figure S4.

**Figure 5:**
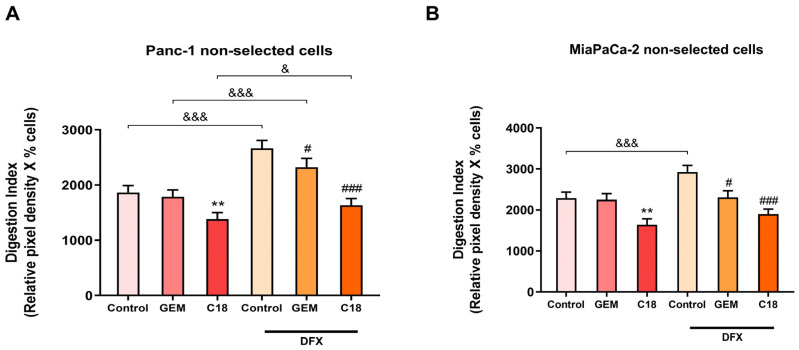
Effect of GEM and C18 on the invadopodia activity of non-pH-selected Panc-1 cells (**A**) and MiaPaCa-2 cells (**B**) in normoxic and hypoxic conditions. C18 is much more effective than GEM in reducing the invadopodia activity of both PDAC cell lines, in the presence and absence of the chemical inducer of hypoxia, DFX (200 µM). The percentage of invadopodia-positive cells and their ECM degradation was determined by fluorescence microscopy. The mean total invadopodia proteolytic activity was then calculated as follows: Digestion Index = percentage of invadopodia-positive cells × mean pixel density of invadopodia/cell. Error bars indicate mean ± S.E.M. (*n* = 3). ** *p* < 0.01 when compared with control; # *p* < 0.05; ### *p* < 0.001 when compared with DFX group; & *p* < 0.05; &&& *p* < 0.001 when compared within the same treatment between the presence or absence of hypoxia.

As shown in Figure 5, in contrast with GEM, which slightly reduced the Digestion Index of the two cell lines only in hypoxic conditions, C18 was highly effective in reducing the Digestion Index in the two non-selected cell lines, both in normoxic and hypoxic conditions. This inhibition in the Digestion Index was due to a reduction in the mean proteolytic activity/cell of both cell lines rather than the percentage of positive cells (Supplementary Figure S5).

### 3.6. C18 Is More Effective than GEM in Counteracting the DFX-Induced Hypoxic Stimulation of Proteolytic Activity and Drug Resistance in pH-Selected Cells

Considering that the acidic-selected cells were endowed with enhanced abilities to digest the ECM under both acidic and hypoxic conditions, we next evaluated whether these typically altered TME conditions could affect the ECM proteolytic response of these cells to the drugs. For this, the two PDAC pH-selected cell lines were cultured in the absence and presence of hypoxia and exposed overnight to 50 µM of both drugs (GEM and C18).

**Figure 6:**
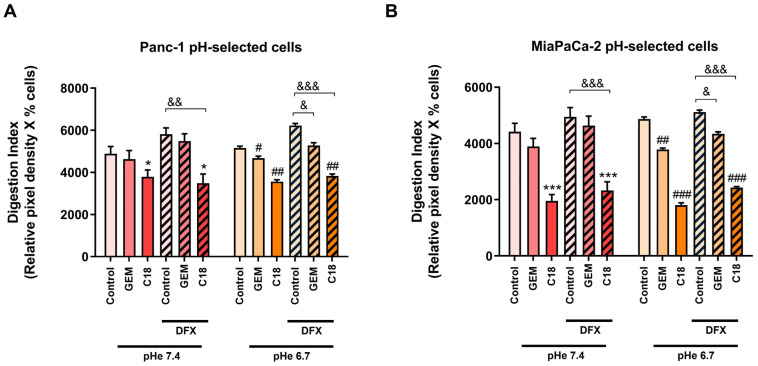
Effect of both GEM and C18 on the invadopodia activity of Panc-1 pH-selected cells (**A**) and MiaPaCa-2 pH-selected cells (**B**) in normoxic and DFX (200 µM)-induced hypoxic conditions. Both GEM and C18 were used at the dose of 50 µM. C18 had a stronger effect in inhibiting invadopodia proteolytic activity of pH-selected cells even under hypoxic and acidic conditions. The percentage of cells forming invadopodia and their ECM degradation were determined by fluorescence microscopy. The mean total invadopodia proteolytic activity was then calculated as follows: Digestion Index = percentage of invadopodia-positive cells × mean pixel density of invadopodia/cell. Error bars indicate mean ± S.E.M. (*n* = 3). * *p* < 0.05; *** *p* < 0.001 when compared with control pH_e_ 7.4; # *p* < 0.05; ## *p* < 0.01; ### *p* < 0.001 when compared with control pH_e_ 6.7; & *p* < 0.05; && *p* < 0.001; &&& *p* < 0.001 when compared within the same pH_e_ between normoxic and hypoxic condition.

We found that in both Panc-1 (Figure 6A) and MiaPaCa-2 (Figure 6B) pH-selected cells, GEM was effective only at pH_e_ 6.7, under both normoxic and hypoxic conditions. On the contrary, C18 was much more effective than GEM in all the experimental conditions. Indeed, C18 significantly reduced the Digestion Index of acid-selected cells at both pH_e_s, under both normoxic and hypoxic conditions. Moreover, C18 was even more effective in the MiaPaCa-2 pH-selected cells than in the Panc-1 pH-selected cells. While hypoxia negatively affected the action of GEM, within the same pH_e_, C18 remained very efficient in reducing the invadopodia proteolytic activity of PDAC cells even under hypoxic conditions. Interestingly, this inhibition in the Digestion Index of both cell lines by C18 was due to both the reduction in the percentage of positive cells and the inhibition of the mean proteolytic activity/cell, while for GEM, the reduction in the mean proteolytic activity/cell was more important (Supplementary Figure S6).

## 4. Discussion

One of the most important hallmarks of PDAC is its rapid progression and metastatic dissemination to the surrounding tissues [29,30]. Indeed, the cause of death for most of PDAC patients is not the primary tumor but the metastatic disease [31]. Metastatic progression involves the successful completion of successive steps, in which the invasion of cancer cells through the ECM is crucial and one of the first events [32]. Invadopodia formation is a process associated with early metastasis, where the invasive cells are able to release matrix proteases and degrade the ECM in response to mechanical and/or chemical cues [33]. Moreover, invadopodia initiation and activity seems to be modulated by TME-derived signals, including the ECM composition (content of Collagen, laminin, and fibronectin), the interaction with stroma cells (especially with activated fibroblasts), matrix stiffness (namely tension and viscosity), and the presence of specific metabolic conditions (extracellular acidosis and hypoxia) [34,35]. To complete the metastatic cascade, invasive cancer cells need to face these challenging events and undergo adaptation to the different microenvironmental contexts through reversible changes, often associated with EMT [36].

The tumor microenvironment plays a crucial role in promoting migration and invasion of cancer cells by either cross-talking with the stroma cells or by providing cancer cells with the ideal physicochemical properties (ECM composition, pH, O_2_, nutrients) to support their energetic and metabolic demands during invasion [13]. Similar to other cancers, PDAC is characterized by a pronounced acidic microenvironment resulting from abnormal blood perfusion and metabolic reprogramming, allowing cancer cells to obtain the necessary energy and nutrients to sustain their uncontrolled proliferation and invasion [12]. Therefore, in order to mimic the features of TME, in this study we used an established model of PDAC cell lines that were adapted to the acidic environment (pH_e_ 6.6) [26] to compare their invasive behavior with the non-selected PDAC cells. While previous studies demonstrated that long-term acidic exposure induces acid adaptation in PDAC cells [37,38,39,40], including in 3D cultures [40], no experiments were conducted in these models to study the differences in the chemotherapeutic resistance of the non-selected vs. selected cells under different TME conditions (e.g., hypoxia and acidosis).

Currently available cytotoxic therapies for PDAC are modestly effective. The main chemotherapy agents are FOLFIRINOX (fluorouracil, oxaliplatin, irinotecan, leucovorin) for individuals with high functional status or gemcitabine (GEM), alone or combined with Nab-Paclitaxel, for individuals with poorer functional status [41]. However, even if over the last decades GEM, alone or more recently in combination, has been utilized as the first-line gold-standard treatment for most PDAC patients [16], it is quickly deaminated in blood, liver, kidney, and other tissues, resulting in a very short half-life [42]. Increasing GEM concentration to overcome its low availability in the target tissue is not an option due to the associated toxicity [43]. Hence, different approaches have been tried to improve GEM’s stability, bioavailability, and in vivo cytotoxic activity. Among them, the synthesis of an acyl moiety that protects the drug from its rapid inactivation improves its antitumor activity compared to the pure drug [19]. The lipophilic amide prodrug of GEM, C18, is emerging as a possible alternative for cancer treatment, since it already exhibited enhanced antitumor activity compared to GEM on human colorectal adenocarcinoma (HT-29) [44], nasopharyngeal carcinoma (KB 396p) [44], and pancreatic ductal adenocarcinoma cells (Panc-1 and their derived stem cells) [22]. However, the efficacy of this prodrug in inhibiting the invasive process and the influence of the principal physical properties of the TME (acidosis and hypoxia) on its anticancer activity is still unknown.

The advances in the 3D cell culture platforms have contributed to improve the study of cellular mechanisms and screening of the efficacy of anticancer therapeutics under in vivo-like conditions [45,46]. Thus, in this study, we used a 3D organotypic cell culture model of pancreatic cancer cells growing at both physiological pH_e_ (pH_e_ 7.4) and acidic pH_e_ (pH_e_ 6.7) on an ECM constituted of a mix of Matrigel and Collagen I, which efficiently mimics the surrounding microenvironment, where the tumor cells are mainly exposed during PDAC in vivo development and progression. On these platforms, we grew two different PDAC cell lines, Panc-1 and the MiaPaCa-2 cells, which were either cultured at pH_e_ 7.4 or exposed for 1 month at pH_e_ 6.6 (pH_e_-selected cells). This is the first analysis of the TME’s impact on the effect of C18 in inhibiting tumor growth and the invasive process in 3D organotypic cultures of PDAC cells.

Here, we observed that acid-selected Panc-1 cells, in line with their extreme resistance to stress metabolic conditions [47] and chemotherapeutics, had a decreased basal cell death compared to the non-selected cells at both pH_e_ 6.7 and 7.4, while acid selection in MiaPaCa2 cells increased basal death compared to the control cells at both pH_e_s (Figure 2). These results are also in accordance with other studies, which demonstrated that Panc-1 cells exhibit higher stemness features than MiaPaCa-2 cells [48]. In line with this, we have already demonstrated that C18 was much more effective than GEM in reducing the growth of both Panc-1 parenchymal cells and their derived cancer stem cells (CSCs), in both Matrigel-rich and Collagen I-rich ECM at the physiological pH_e_ of 7.4 [22]. In this study, we show that C18 not only was more cytotoxic than GEM in physiological conditions but was also more efficient in inhibiting cell viability (Figure 1) and inducing cell death of both Panc-1 and MiaPaCa-2 cell lines at pH_e_ 6.7 (Figure 2). Therefore, the C18 treatment was more effective than GEM in both acid-selected and control cell lines in all culture conditions (Figure 1 and Figure 2). To assess possible alterations to gemcitabine therapeutic targets by acidosis, we interrogated the same transcriptional dataset presented in [26] and found that two of the three targets annotated by the DrugBank knowledgebase as gemcitabine-specific (namely RRM1, TYMS, CMPK1) are significantly downregulated by acidosis ([49] (go.drugbank.com, accession DB00441; accessed on 22 March 2024). Specifically, RRM1 showed a log2FC = −1.49 (adj. *p*-value = 3.2 × 10^−4^), while TYMS had s log2FC = −1.67 (adj. *p*-value = 3.9 × 10^−4^), overall suggesting a possible impairment of some mechanism of action of gemcitabine. To further investigate the transcriptional aspects related to drug resistance acquired by Panc-1 cells under acidosis, we then tested the list of differentially expressed genes for the overrepresentation of gene sets involved in some known drug-resistance mechanisms as annotated by the DRESIS ([50], dresis.idrblab.net) and DRMref ([51] ccsm.uth.edu/DRMref) databases. Among these, the UAPP (i.e., the “Unusual Activation of Pro-survival Pathway”) was the most significantly enriched mechanism of drug resistance (fold enrichment = 2.14, *p*-value = 5.1 × 10^−8^, hypergeometric test), also further confirming the results of the functional enrichment analysis already reported in [26]. These data could possibly explain the increased activity of C18.

Further, this C18-induced cytotoxicity in the two cell lines and at the two pH_e_s is an important requisite for a candidate antimetastasis drug, which should not only prevent metastasis or slow down further dissemination after initial local invasion has occurred but also reduce the already established (micro)metastases by inducing cell death in the metastatic cells. The differences observed in the effects of acidic pH_e_ and of GEM and C18 activity on cell viability and death could be explained by the different loss of function p53 mutations occurring in the two PDAC cell lines analyzed, the Panc-1 (R273H) and MiaPaCa-2 (R248W) cells. Indeed, p53 is known to regulate the response to diverse cellular stress events by inducing cell cycle arrest, apoptosis and senescence and modifying the tumor stroma with consequent changes in GEM resistance [52], and a crosstalk between tumor acidosis, ECM, and p53 expression has been reported to increase growth in 3D PDAC cell cultures [40].

However, as mentioned before, the major concern with PDAC is the very early occurrence of metastasis, which is responsible for the nearly half a million cancer deaths globally each year from PDAC and is extremely difficult to treat. Indeed, while most chemotherapeutics currently used for PDAC have a cytotoxic effect, there are still very few treatment options for blocking metastasis [53]. Therefore, a candidate drug for PDAC treatment should be effective not only in reducing tumor growth but also in inhibiting the processes associated with invasion and metastasis. In this study, we assessed (i) whether the TME may influence the invadopodial proteolytic activity of both PDAC cell lines and (ii) the efficacy of both GEM and C18 on the invadopodia activity of these cells under physiological and acidic/hypoxic microenvironmental stress conditions.

Interestingly, the acute extracellular acidosis, which is commonly associated with invasion and metastasis [54], decreased the ECM’s digestion capacity (invadopodia activity) in control, non-acid-selected PDAC cells (Figure 3). A possible explanation for this is that normal pancreatic cells in healthy tissue are often exposed to cyclic waves of acidic pH_e_, depending on the time of the last meal [55]. Therefore, pancreatic cells could be less sensitive to acidic pH_e_ than cells residing in other tissues, such as breast and prostate, and an acute exposure of non-acid-selected PDAC cells to an acidic pH_e_ may not be sufficient to shift their behavior towards a more invasive phenotype. However, when the acid-selected cells were exposed to acidic extracellular conditions, they increased their invadopodial ECM proteolytic activity in comparison to cells grown at pH_e_ 7.4 (Figure 4). This suggests that PDAC cells can (re-)acquire their proteolytic response/phenotype to an acidic TME when they have had the time to adapt to an acidic extracellular environment. A similar pattern of invasive capacity across a Matrigel layer for these cell lines has been recently reported [26]. On the other hand, we found that treating the cells with DFX, a chemical inducer of hypoxia, increased the ECM digestion capacity of both control and acid-selected PDAC cells at both pH_e_s (Figure 3 and Figure 4). Moreover, when both extracellular acidosis and hypoxia were present, we observed an additive effect in increasing the ability of the acid-selected PDAC cells to degrade the ECM in order to invade the surrounding tissue (Figure 4). These findings support the hypothesis that pancreatic cancer cells may need a pre-exposure to the acidic microenvironment before they acquire the ability to initiate the metastatic cascade, and this is independent of their response to hypoxia.

Most importantly, we found that C18 was also much more efficient in inhibiting invadopodial ECM proteolysis than GEM under both normoxic and hypoxic conditions and in the presence or absence of extracellular acidosis (Figure 5 and Figure 6). Further, C18’s inhibition of invadopodial ECM proteolysis was cell line-independent as it was very similar in both Panc-1 and MiaPaCa-2. Therefore, the well-known TME barriers to therapy, such as hypoxia and acidosis, seem to not significantly affect the efficiency of C18 as an anti-invasion treatment and strongly suggest that C18 might be a valid alternative for GEM in anticancer and especially anti-metastatic therapy for PDAC.

## Data Availability

The data are available in this article and Supplementary Material.

## Supplementary Materials

The following supporting information can be downloaded at: https://www.mdpi.com/article/10.3390/cells13090730/s1, Figure S1A. Stability of GEM in RMPI 1640 at pH 5,0, 6,7, 7,4 over 96 hours. % of unmodified. Figure S1B. Stability of C18GEM prodrug in RMPI 1640 at pH 5,0, 6,7, 7,4 over 96 hours. % of unmodified compound vs time (hours). compound vs time (hours). Figure S2. Effect of both hypoxia and acidic pH_e_ on the number of invadopodia-positive cells and their ECM-proteolytic activity/cell in non acid selected Panc-1 cells (A) and MiaPaCa-2 cells (B). The percentage of cells that formed invadopodia and mean ECM degradation/cell was determined by fluorescence microscopy. Error bars indicate mean ± S.E.M (*n* = 3). * *p* < 0.05; *** *p* < 0.001 when compared with control pH_e_ 7.4; # *p* < 0.05; ## *p* < 0.01 when compared with control pH_e_ 6.7; & *p* < 0.05; && *p* < 0.01 when compared within the same treatment between the two different pH_e_. Figure S3. Effect of both hypoxia and acidic pH_e_ on the number of invadopodia-positive cells and their ECM-proteolytic activity/cell in Panc-1 pH-selected cells (A) and MiaPaCa-2 pHselected cells (B). The percentage of invadopodia positive cells and their mean ECM degradation/cell was determined by fluorescence microscopy. Error bars indicate mean ± S.E.M (*n* = 3). * *p* < 0.05; *** *p* < 0.001 when compared with control pH_e_ 7.4; # *p* < 0.05; ### *p* < 0.001 when compared with control pH_e_ 6.7; & *p* < 0.05 && *p* < 0.01 when compared within the same treatment between the two different pH_e_. Figure S4. Effect of GEM and C18 on Invadopodia in normoxia and hypoxia. These panels show typical images related to the experiments of Figure 5 of proteolytic digestion in green and actin in red. The white bar in the upper right (CTRL) panel of each cell line represents 50 μm. Figure S5. Effect of GEM and C18 on the number of invadopodia-positive cells and their ECMproteolytic activity/cell in non pH-selected Panc-1 cells (A) and MiaPaCa-2 cells (B) in normoxic and hypoxic conditions. The percentage of invadopodia positive cells and their mean ECM degradation/cell was determined by fluorescence microscopy. Error bars indicate mean ± S.E.M (*n* = 3). * *p* < 0.05; ** *p* < 0.01 when compared with control; ## *p* < 0.01 when compared with DFX group; & *p* < 0.05 when compared within the same treatment between the presence or absence of hypoxia. Figure S6. Effect of both GEM and C18 on the number of invadopodia-positive cells and their ECM-proteolytic activity/cell in Panc-1 pH-selected cells (A) and MiaPaCa-2 pH-selected cells (B) in normoxic and DFX (200 μM)-induced hypoxic conditions. The percentage of cells forming invadopodia and their ECM degradation were determined by fluorescence microscopy. Error bars indicate mean ± S.E.M (*n* = 3). * *p* < 0.05; ** *p* < 0.01; *** *p* < 0.001 when compared with control pH_e_ 7.4; # *p* < 0.05; ### *p* < 0.001 when compared with control pH_e_ 6.7; & *p* < 0.05; && *p* < 0.001; &&& *p* < 0.001 when compared within the same pH_e_ between normoxic and hypoxic condition.
