# Supplementary material for: Tumor Microenvironment Modulates Invadopodia Activity of Non-Selected and Acid-Selected Pancreatic Cancer Cells and Its Sensitivity to Gemcitabine and C18-Gemcitabine
Source: Cells. 2024 Apr 23;13(9):730. doi: 10.3390/cells13090730 (PMC11083398; doi:10.3390/cells13090730)
Supplement: Supplementary file 1 [file cells-13-00730-s001.zip › Supplementary Figures revision.pdf]

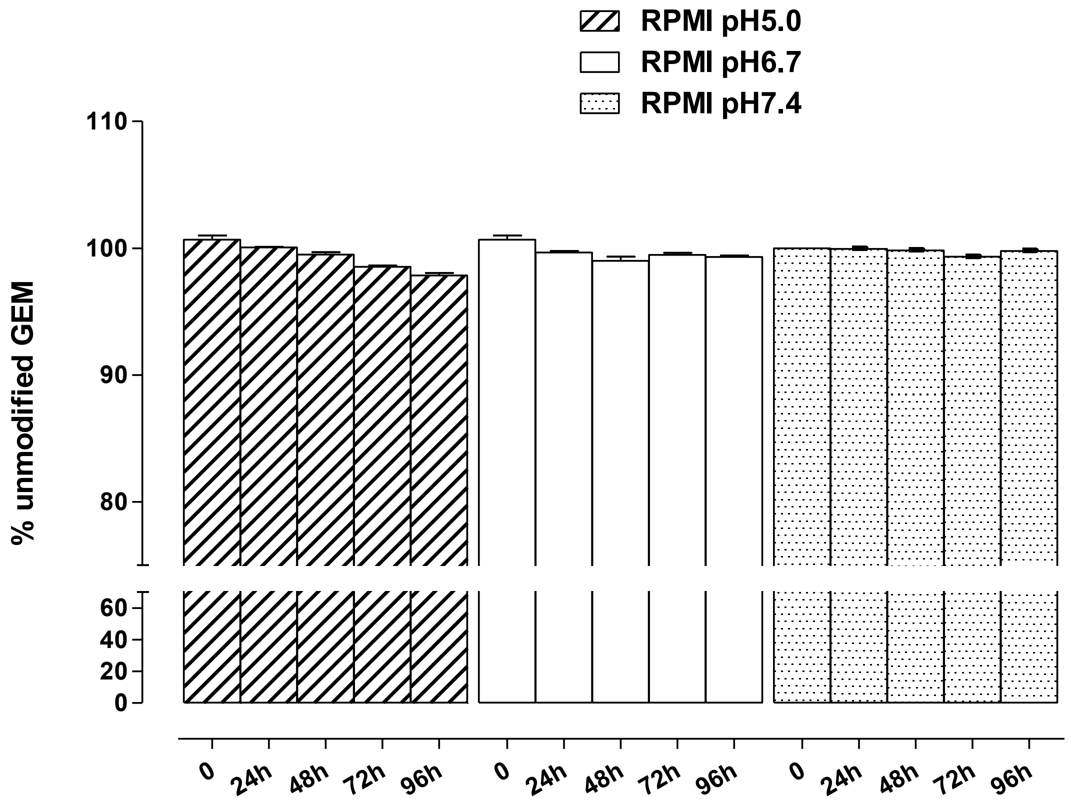

Figure S1A. Stability of GEM in RMPI 1640 at pH 5,0, 6,7, 7,4 over 96 hours. % of unmodified compound vs time (hours).

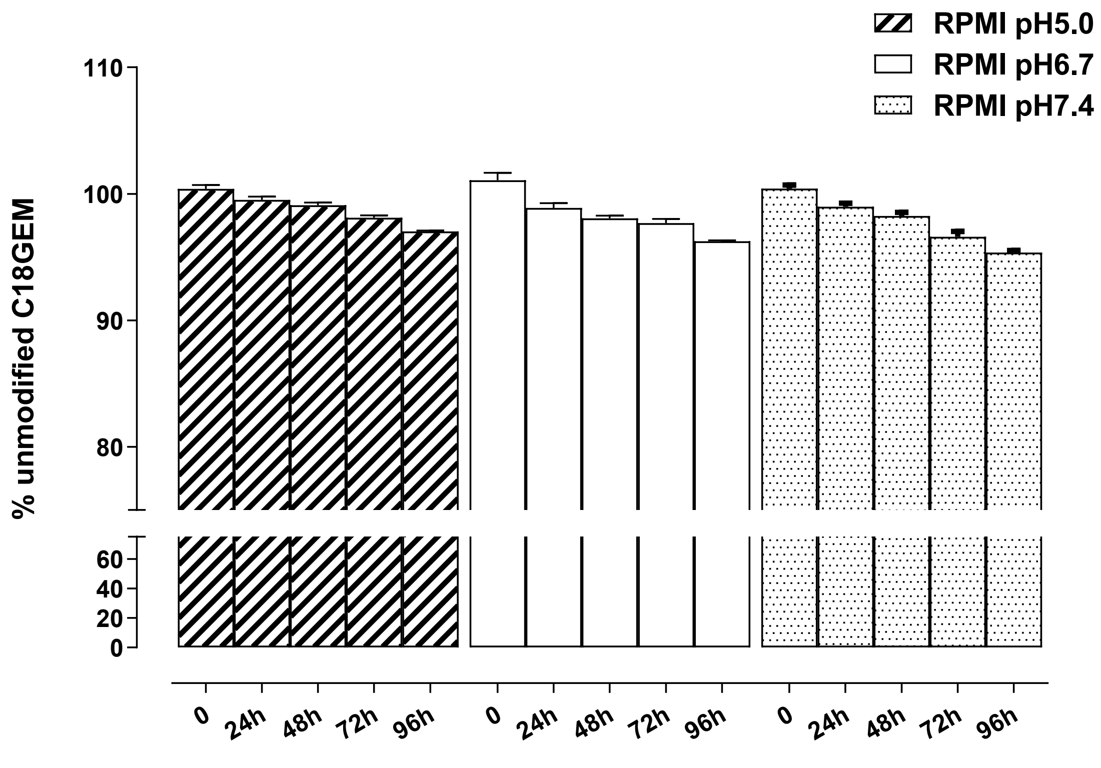

S1B. Stability of C18GEM prodrug in RMPI 1640 at pH 5,0, 6,7, 7,4 over 96 hours. % of unmodified compound vs time (hours).

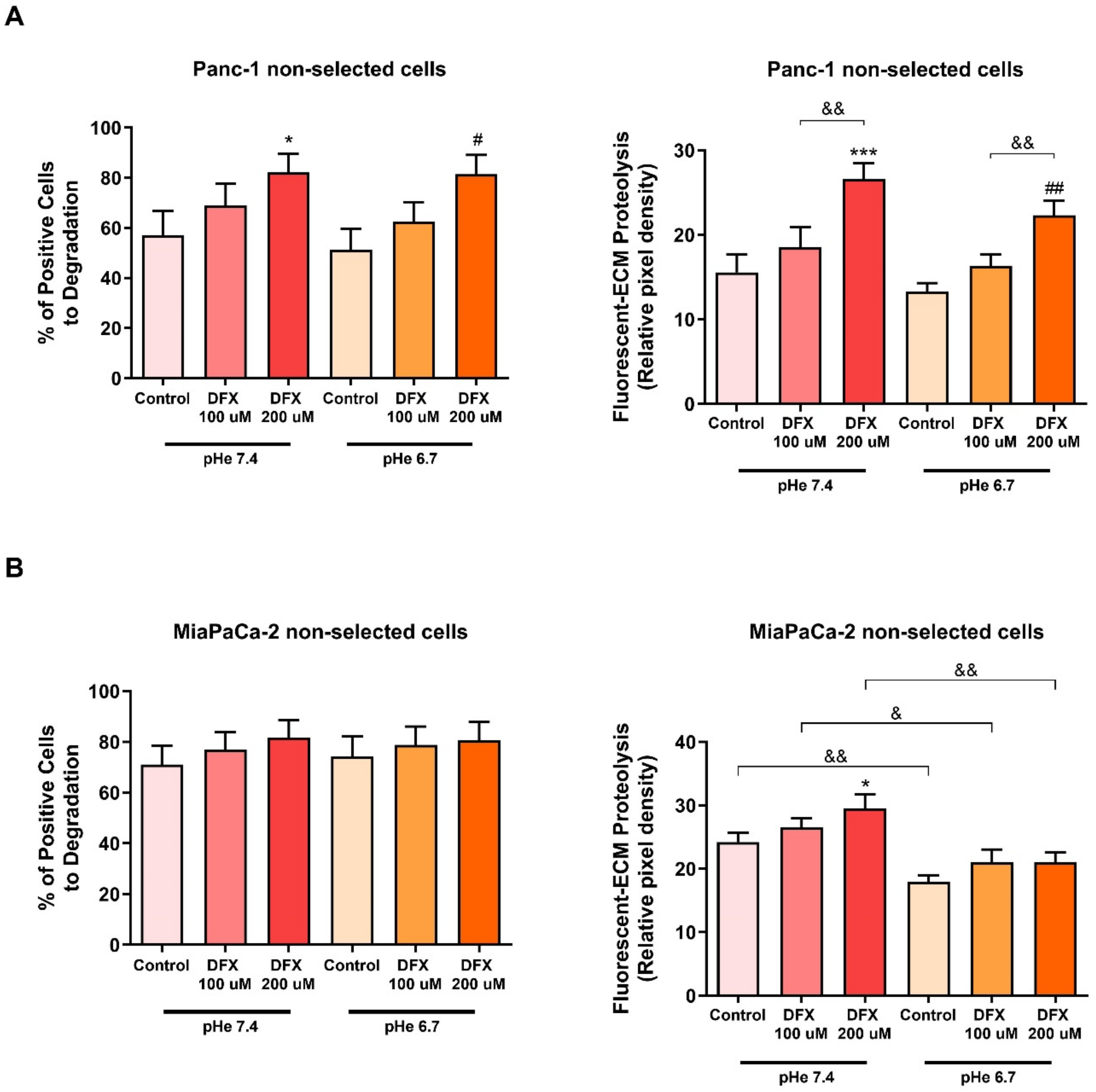

Figure S2. Effect of both hypoxia and acidic pH_e on the number of invadopodia-positive cells and their ECM-proteolytic activity/cell in non acid selected Panc-1 cells (A) and MiaPaCa-2 cells (B). The percentage of cells that formed invadopodia and mean ECM degradation/cell was determined by fluorescence microscopy. Error bars indicate mean ± S.E.M (n=3). * $p<0.05$; *** $p<0.001$ when compared with control pH_e 7.4; # $p<0.05$; ## $p<0.01$ when compared with control pH_e 6.7; & $p<0.05$; && $p<0.01$ when compared within the same treatment between the two different pH_e.

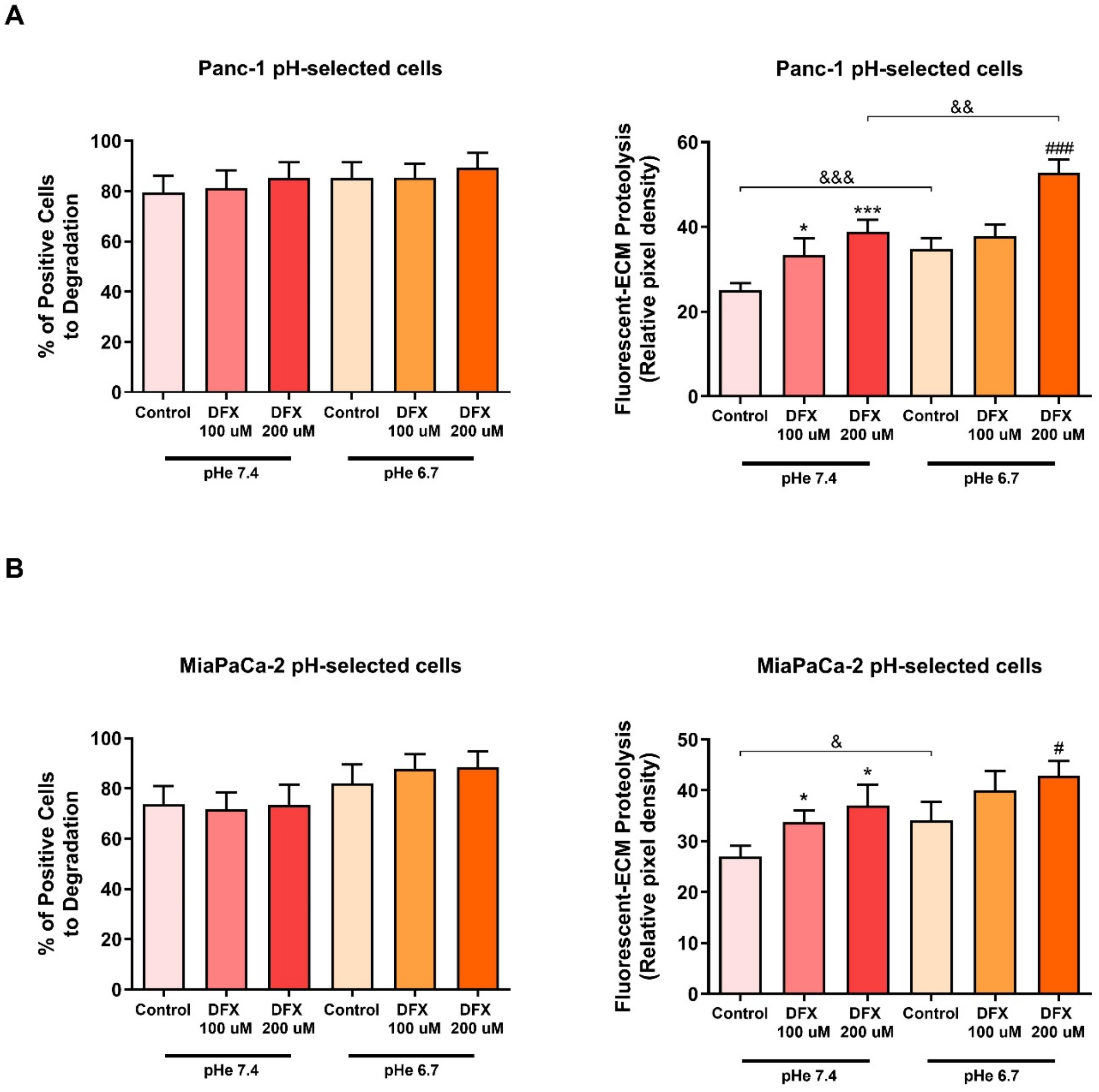

Figure S3. Effect of both hypoxia and acidic pHe on the number of invadopodia-positive cells and their ECM-proteolytic activity/cell in Panc-1 pH-selected cells (A) and MiaPaCa-2 pH-selected cells (B). The percentage of invadopodia positive cells and their mean ECM degradation/cell was determined by fluorescence microscopy. Error bars indicate mean ± S.E.M (n=3). * p<0.05; *** p<0.001 when compared with control pH_e 7.4; # p<0.05; ### p<0.001 when compared with control pH_e 6.7; & p<0.05 && p<0.01 when compared within the same treatment between the two different pH_e.

Figure S4. Effect of GEM and C18 on Invadopodia in normoxia and hypoxia. These panels show typical images related to the experiments of Figure 5 of proteolytic digestion in green and actin in red. The white bar in the upper right (CTRL) panel of each cell line represents 50µm.

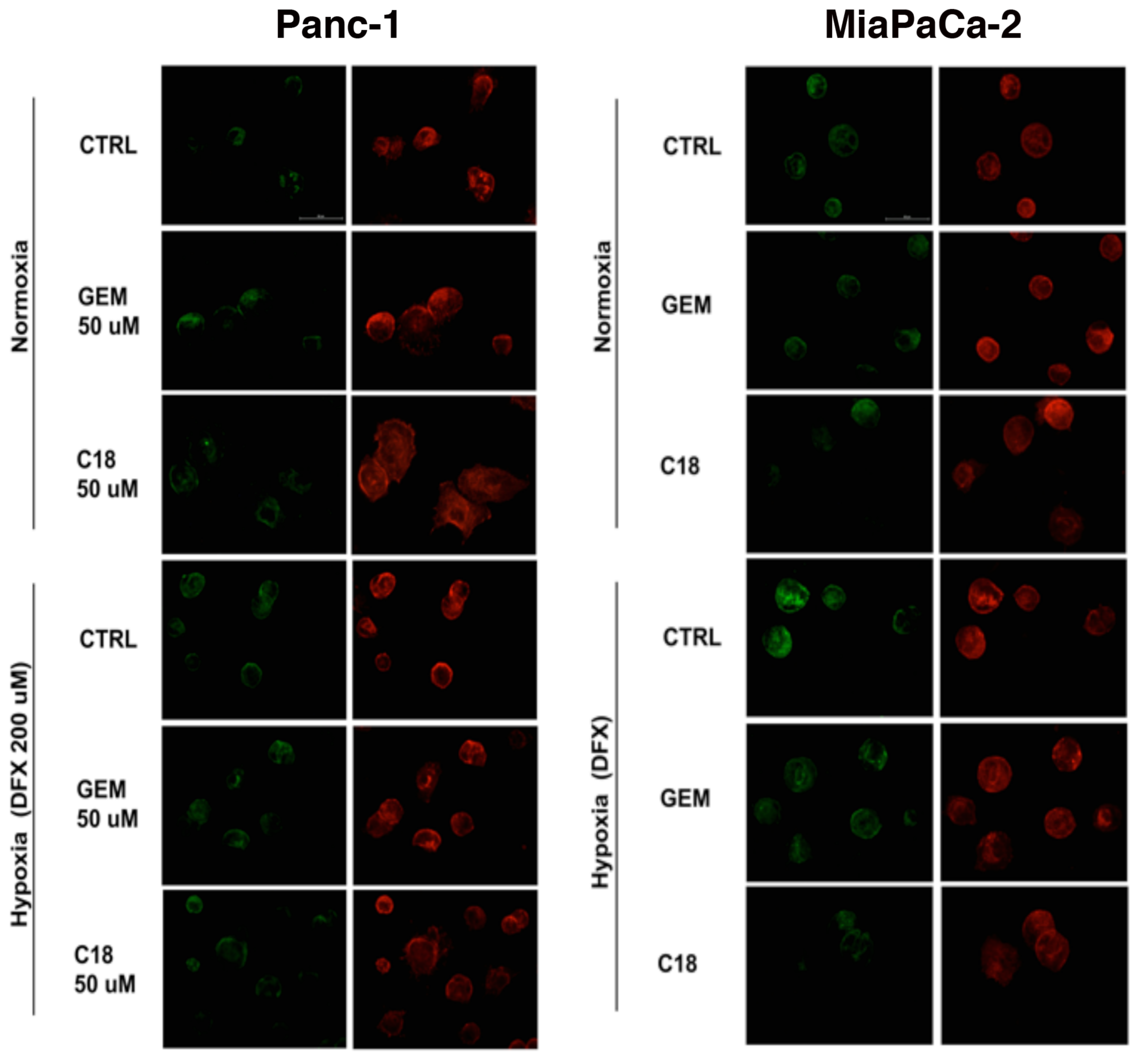

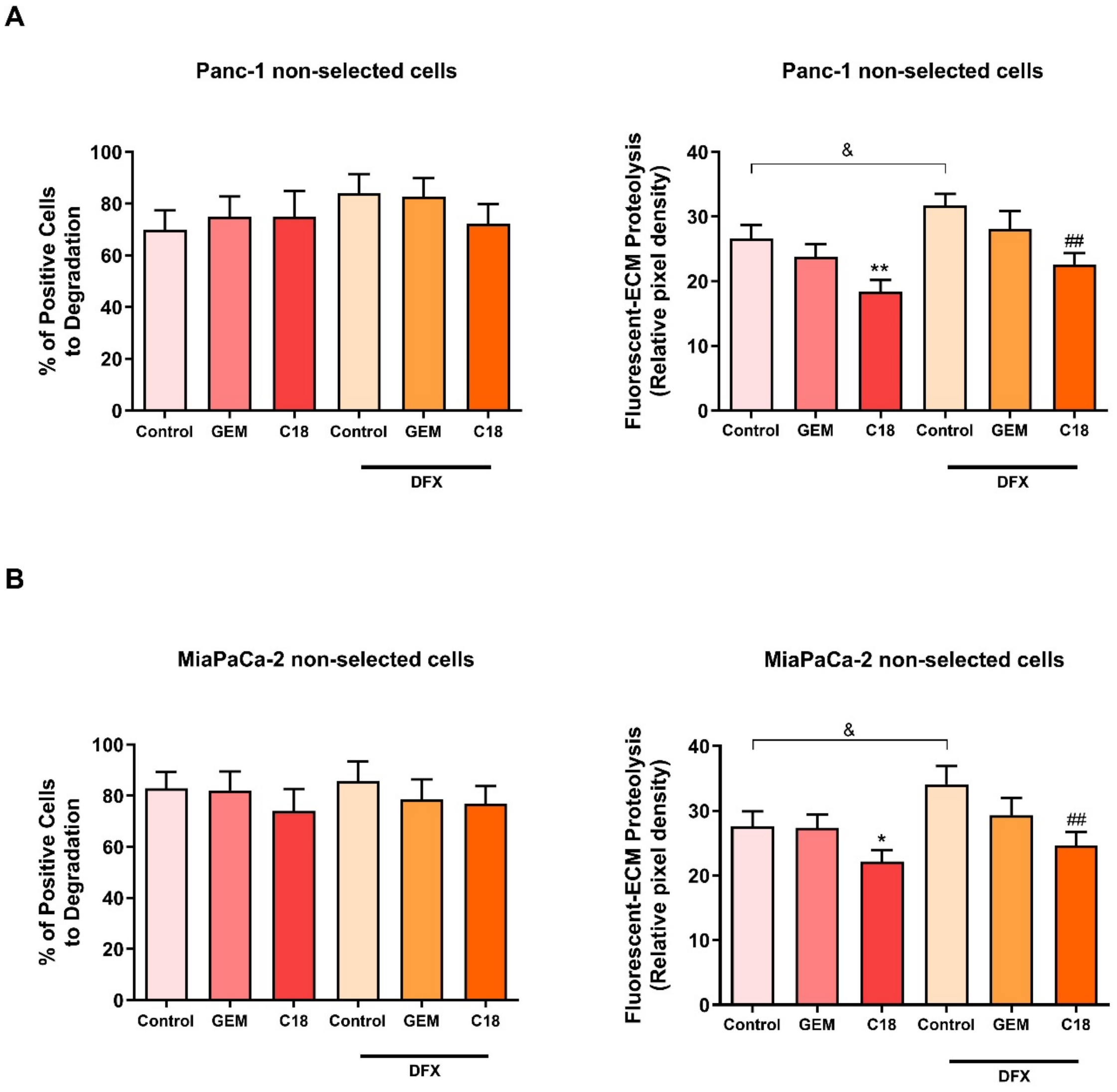

Figure S5. Effect of GEM and C18 on the number of invadopodia-positive cells and their ECM-proteolytic activity/cell in non pH-selected Panc-1 cells (A) and MiaPaCa-2 cells (B) in normoxic and hypoxic conditions. The percentage of invadopodia positive cells and their mean ECM degradation/cell was determined by fluorescence microscopy. Error bars indicate mean ± S.E.M (n=3). * $p<0.05$; ** $p<0.01$ when compared with control; ## $p<0.01$ when compared with DFX group; & $p<0.05$ when compared within the same treatment between the presence or absence of hypoxia.

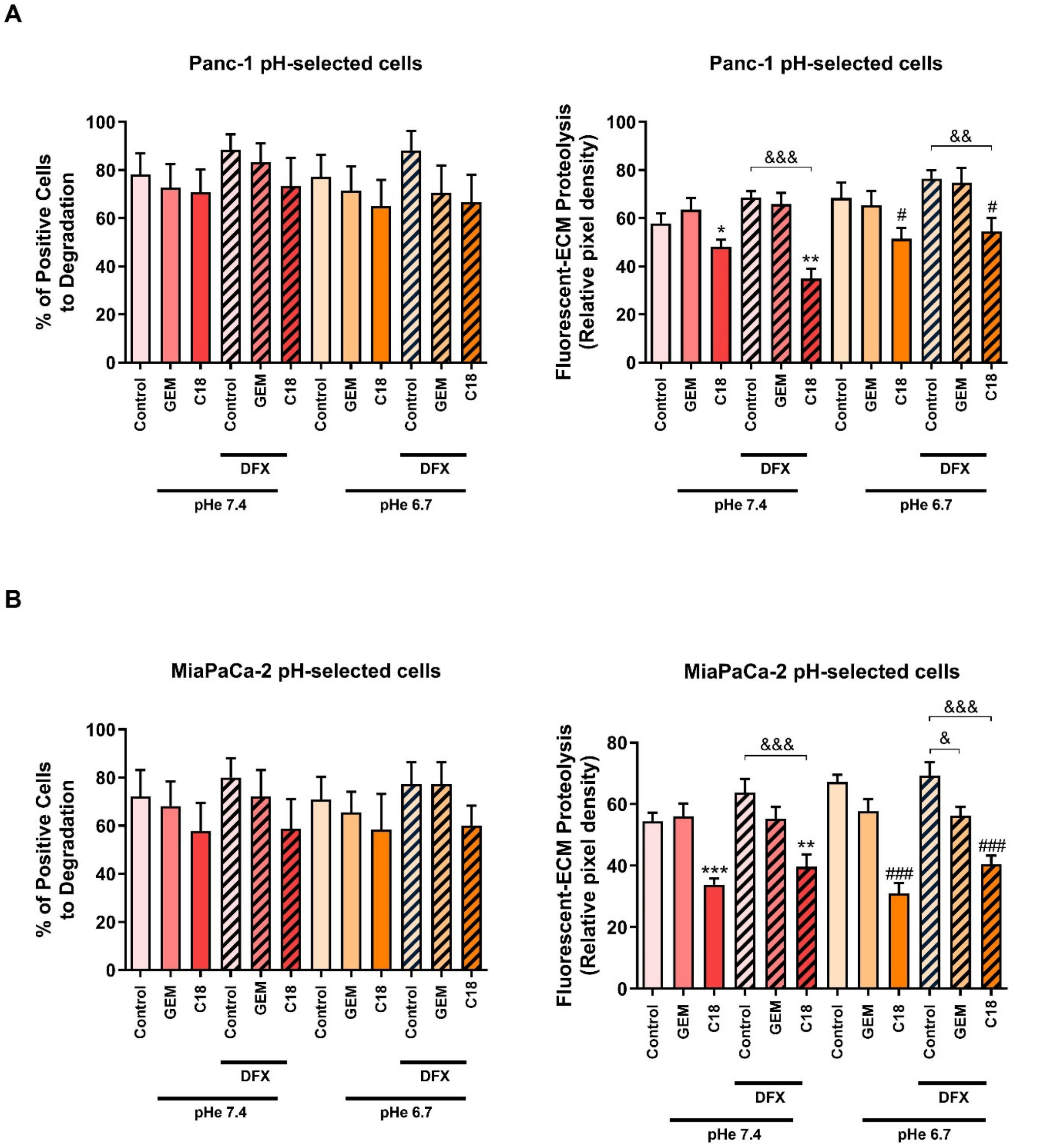

Figure S6. Effect of both GEM and C18 on the number of invadopodia-positive cells and their ECM-proteolytic activity/cell in Panc-1 pH-selected cells (A) and MiaPaCa-2 pH-selected cells (B) in normoxic and DFX (200µM)-induced **hypoxic conditions.** The percentage of cells forming invadopodia and their ECM degradation were determined by fluorescence microscopy. Error bars indicate mean ± S.E.M (n=3). * p<0.05; ** p<0.01; *** p<0.001 when compared with control pH_e 7.4; # p<0.05; ### p<0.001 when compared with control pH_e 6.7; & p<0.05; && p<.001; &&& p<0.001 when compared within the same pH_e between normoxic and hypoxic condition.
